# Environmental and Geographical Factors Structure Soil Microbial Diversity in New Caledonian Ultramafic Substrates: A Metagenomic Approach

**DOI:** 10.1371/journal.pone.0167405

**Published:** 2016-12-01

**Authors:** Véronique Gourmelon, Laurent Maggia, Jeff R. Powell, Sarah Gigante, Sara Hortal, Claire Gueunier, Kelly Letellier, Fabian Carriconde

**Affiliations:** 1 Institut Agronomique néo-Calédonien (IAC), Axe 2 “Diversités biologique et fonctionnelle des écosystèmes terrestres”, Nouméa, New Caledonia; 2 CIRAD, UMR AGAP, Nouméa, New Caledonia; 3 Hawkesbury Institute for the Environment, Western Sydney University, Penrith, New South Wales, Australia; 4 Société Le Nickel (SLN) – Groupe ERAMET, Département Environnement, Nouméa, New Caledonia; University of Milan, ITALY

## Abstract

Soil microorganisms play key roles in ecosystem functioning and are known to be influenced by biotic and abiotic factors, such as plant cover or edaphic parameters. New Caledonia, a biodiversity hotspot located in the southwest Pacific, is one-third covered by ultramafic substrates. These types of soils are notably characterised by low nutrient content and high heavy metal concentrations. Ultramafic outcrops harbour diverse vegetation types and remarkable plant diversity. In this study, we aimed to assess soil bacterial and fungal diversity in New Caledonian ultramafic substrates and to determine whether floristic composition, edaphic parameters and geographical factors affect this microbial diversity. Therefore, four plant formation types at two distinct sites were studied. These formations represent different stages in a potential chronosequence. Soil cores, according to a given sampling procedure, were collected to assess microbial diversity using a metagenomic approach, and to characterise the physico-chemical parameters. A botanical inventory was also performed. Our results indicated that microbial richness, composition and abundance were linked to the plant cover type and the dominant plant species. Furthermore, a large proportion of Ascomycota phylum (fungi), mostly in non-rainforest formations, and Planctomycetes phylum (bacteria) in all formations were observed. Interestingly, such patterns could be indicators of past disturbances that occurred on different time scales. Furthermore, the bacteria and fungi were influenced by diverse edaphic parameters as well as by the interplay between these two soil communities. Another striking finding was the existence of a site effect. Differences in microbial communities between geographical locations may be explained by dispersal limitation in the context of the biogeographical island theory. In conclusion, each plant formation at each site possesses is own microbial community resulting from multiple interactions between abiotic and biotic factors.

## Introduction

Soil microorganisms play crucial roles in ecosystems functioning [1], such as in biogeochemical cycles, soil stability, plant growth and plant community dynamics [2–5]. One gram of soil can harbour millions to billions of bacteria, hundreds of meters of fungal hyphae, and large species diversity [6]. Soil microorganism communities have been shown to be influenced by abiotic and biotic factors [7–10]. Among the edaphic parameters, pH is the factor that most strongly influences soil bacterial communities [9,11]. Some studies have also shown that phosphorus [9,12] and soil texture [13] can shape soil bacterial communities. The effects of abiotic parameters have been less often demonstrated in fungal communities [10,14]. Regarding biotic factors, several studies have clearly shown that aboveground plant cover influences the fungal community structure and the functional diversity of forest soils [15–17]. However, plant community composition has little to no effect on bacterial communities [14,18]. Furthermore, soil bacteria and fungi have also been known to interact with each other. Some bacteria, called Mycorrhiza Helper Bacteria (MHB), improve the development of mycorrhiza and are fungal specific [19,20]. Fungi are able to excrete compounds that attract different bacterial taxa according to their chemical composition [21,22]. Nevertheless, despite the large diversity and the significance of these microorganisms to terrestrial ecosystem processes, factors influencing their richness, composition and abundance remain largely unknown.

To date, most studies on soil microbial diversity have been performed in temperate regions and little research has been undertaken in tropical and subtropical regions [23]. New Caledonia is a subtropical archipelago located in the southwestern Pacific, between 20° and 23° South latitude and 164° and 167° east longitude. In this territory, the endemism rate in vascular plants is approximately 74% [24,25]. Taking the land surface into consideration, New Caledonia exhibits the world’s highest plant endemism richness [26]. However, this diversity is strongly threatened by bushfire and mining activities [27]. Due to this high endemism rate and anthropogenic threats, Myers *et al*. [28] identified New Caledonia as a biodiversity hotspot, *i*.*e*., a priority conservation area. This high rate of endemism is due to different factors, such as the geographical isolation of the archipelago and its particular geological history, which has led to a mosaic of soils. The island is dominated by three types of substrate: (1) ultramafic substrates (c.a. 30% surface coverage); (2) volcano-sedimentary substrates (c.a. 48% surface coverage); and (3) calcareous substrates (c.a. 21% surface coverage).

**Fig 1:**
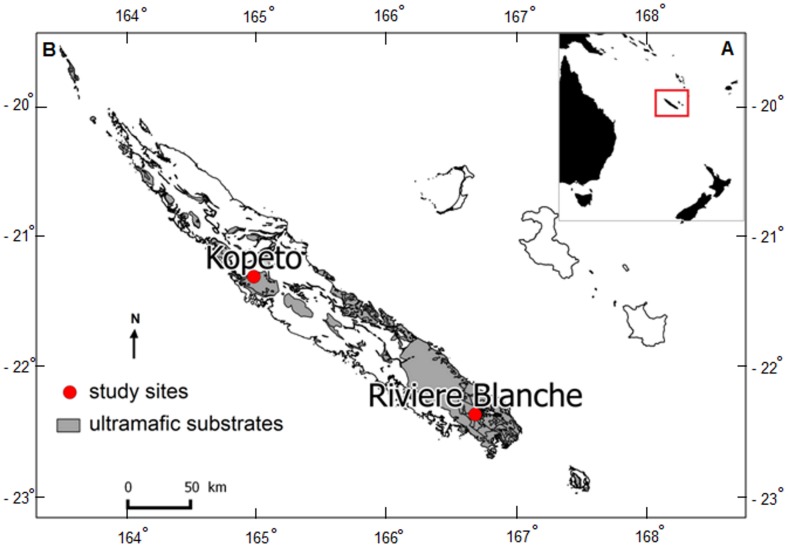
(A) Location of New Caledonia archipelago in the southwest Pacific, and (B) repartition of ultramafic substrates (in grey) in New Caledonia. The two study sites are represented by red dots.

Ultramafic substrates (also called serpentine soils) are mainly characterised by low nutrient availability (*e*.*g*., nitrogen, potassium and phosphorus) and high concentrations of heavy metals (*e*.*g*., nickel, chromium and manganese). These particular substrates have not been found to limit bacterial and fungal diversity [29,30]. In New Caledonia, soils derived from ultramafic rocks are represented by two different types of massif: the ‘Massif du Grand Sud’ and fragmented isolated islets [31]. The ‘Massif du Grand Sud’ forms a continuous southern unit covering 3015 km^2^ of the main island (Fig 1). The second type of ultramafic substrates is situated on the northwestern coast in the form of isolated massifs, representing ultramafic islets surrounded by volcano-sedimentary substrates. Ultramafic outcrops host diverse vegetation types [32]. This vegetation is mainly represented by sclerophyllous shrubland formations, called ‘maquis miniers’, and to a lesser extent by rainforest. Maquis are floristically and physiognomically varied and are often dominated by a single species or few species, in particular by the Myrtaceous plant species that belongs to the genus *Tristaniopsis* [32]. These plant formations mostly represent secondary vegetation resulting from the degradation of the initial rainforests [33]. The rainforests have been fragmented and currently occupy restricted areas (20% of ultramafic surface). New Caledonian rainforests encompass a large array of formation types, from mixed rainforests to monodominant stands, *i*.*e*., forests dominated at the canopy layer by one species, such as by the *Nothofagus* (Nothofagaceae) plant species [32,34]. Based on the geographic discontinuity of ultramafic massifs and the existence of different plant formations, habitats on ultramafic outcrops are consequently heterogeneous and could potentially harbour distinct microbial communities. Studies performed in New Caledonia on these types of soils have focused on bacterial and fungal adaptation to nickel [35,36], the effects of mining activities and the restoration of microbial community structure [37], the mycorrhizal status of different plants growing on these substrates [38,39], or again the effects of arbuscular mycorrhizal fungi (AMF) on plant adaptations to nickel-rich substrates [40,41]. However, none of these studies have focused on soil microbial composition on ultramafic substrates in different plant formations and at different sites along New Caledonia.

Due to the characteristics of ultramafic substrates, *i*.*e*., plant composition, heterogeneity and discontinuity of habitats, in this study we tested two hypotheses: (i) environmental factors, *i*.*e*., plant cover and edaphic parameters, have an effect on soil bacterial and fungal community structure; and (ii) the geographic discontinuity of ultramafic massifs in New Caledonia impacts microbial biodiversity. To this end, we worked on two sites on New Caledonian ultramafic substrates. At each site, four plant formations were studied, ranging from a sedge-dominated formation to a well-established mixed rainforest. These represent four stages of a chronosequence that constitute a pattern of plant succession. By using high-throughput sequencing (Illumina MiSeq), the soil microbial diversity associated with each plant formation was assessed. Furthermore, a botanical inventory and soil physico-chemical analysis were also performed on each plant formation and at each site to study relationships between environmental parameters and soil microbial communities.

## Materials and Methods

### Ethics statement

Collection permits were obtained from the 'Province Sud' and 'Province Nord' of New Caledonia.

### Study sites

The Kopéto site is a nickel mine on the Kopéto Massif, a fragmented isolated islet of ultramafic substrate, which is situated on the northwestern portion of the main island (21°10’S-165°0’E), culminating at 1,100 m and receiving an average of 1,200–1,500 mm of rainfall per year. Mining activity occurred first between 1880 and 1983, and was restarted in 1994, remaining active today. The Rivière Blanche site, in the ‘Rivière Bleue’ Provincial Park, has been a reserve since 1980 and is located in the South of New Caledonia, on the ‘Massif du Grand Sud’ (22°9’S-166°41’E). This site has an altitude between 300 and 500 m and an average annual precipitation of 3,000 mm. At the beginning of the 20^th^ century, chromium mines and forestry occurred in this area but have now been stopped.

These two study sites (Fig 1) were selected based on three main criteria: (i) they represent the two possible forms of ultramafic substrates present on the main island, *i*.*e*., fragmented isolated islet and ‘Massif du Grand Sud’; (ii) the presence of the four plant formations of interest that constitute a potential vegetal succession, *i*.*e*., sedge-dominated formation, *Tristaniopsis* spp. maquis, and two types of rainforests: a *Nothofagus aequilateralis* monodominant rainforest, according to Connell and Lowman criteria [42], and a mixed rainforest; and finally, (iii) the presence of the four plant formations of interest with significant abundance to allow installation of our plot system.

### Vegetation types studied using a chronosequence

The four plant formations studied are as follows: (i) sedge-dominated formation (S); (ii) *Tristaniopsis* spp. maquis (Mq); (iii) monodominant *Nothofagus aequilateralis* rainforest (Na); and (iv) mixed rainforest (M) represent different stages of potential vegetal succession.

1. The sedge-dominated formation in this study can be described as a plant formation with vegetation constituted primarily by sedges (Cyperaceae) and ferns, covering between 15 and 80% of the ground.
2. Maquis is an open sclerophyllous shrubland formation, with a top stratum of less than 5 to 6 m [43]. *Tristaniopsis* genus belongs to Myrtaceae and is ubiquitous in maquis. In New Caledonia, this genus is represented by 13 species that are endemic, and some of them have a highly restricted geographical distribution. Six out of the 13 species are known, to date, to be associated with ectomycorrhizal fungi [44].
3. *Nothofagus aequilateralis* is a forest pioneer tree [45]. This species is known to form monodominant stands in rainforests (*i*.*e*., more than 50% of the canopy is represented by this species) [34]. In New Caledonia, five endemic species of *Nothofagus* are present on the mainland. *N*. *aequilateralis* is one of the most common and is found at between 150 and 1,250 m of altitude, with a repartition from the South to the North of the main island [34]. To date, four out of the five species have been characterised as forming ectomycorrhizal symbiosis.
4. A mixed rainforest is defined as a forest that is not dominated in the canopy by a single tree species. Nevertheless, plant species known to form monodominant stands can be present in mixed rainforests, but as isolated trees.

### Study design and soil sampling

At each site, sixteen plots of 20 m x 20 m. Four plots per vegetation types were established. All of the plots were separated from each other by at least 100 m. Nonmetric Multi-Dimensional Scaling conducted on a botanical inventory of each plot indicated that all four plots from the same plant formation were grouped together (S1 Fig). This allowed us to consider them as repetitions for each plant formation. The positioning of plots at the Kopéto and Rivière Blanche sites is shown in S2 Fig. In each plot, a grid was drawn following the design in S3 Fig. Per plot, 25 soils cores of 5 cm diameter and 10 cm depth were sampled at each grid junction and were bulked together to make a composite sample. This sampling design was used to avoid a potential effect from fine-scale spatial heterogeneity of soil microorganisms, and subsequently, to represent the entire plot. These soil samples were conserved in a cool place in the field and then stored at -20°C in the next three hours.

### Botanical inventory

For each plot, plant coverage was assessed using the Braün-Blanquet abundance index [46]. This method measures the percentage of plant coverage by using coefficients ranked from + (representing less than 1% of coverage) to 5 (between 75 and 100% of coverage). Plants were identified for most of them at a species level, with the support of the Herbarium of IRD (NOU) (Nouméa, New Caledonia). For further analysis, epiphytes and liana species were removed from the dataset to retain only species related to the soil.

### Soil physical-chemical analysis

The soil cores were sent to ActLab (Ancaster, Ontario, Canada) for physico-chemical analyses. The soil texture, pH, organic matter, organic carbon, nitrogen (N), ratio calcium/magnesium (Ca/Mg), potassium/magnesium (K/Mg), total elements (iron (Fe), nickel (Ni), silica (Si), aluminium (Al), manganese (Mn), chromium (Cr), cobalt (Co), potassium (K), sodium (Na), calcium (Ca), and magnesium (Mg)), exchangeable elements (sulphur (S), calcium, magnesium, sodium, potassium and aluminium) and total phosphorus (P) were measured in each soil sample. The soil texture (sand, silt and clay percentage) was determined by using a hydrometer. The organic matter content was measured as loss under ignition over 3 hours at 360°C, and the organic carbon content was determined by Infra-Red (IR) detection. The determination of soil total nitrogen was evaluated by combustion, whereas the content of exchangeable soil elements such as K, Ca, Mg and Na were obtained with nitrate extraction. The exchangeable S and Fe were measured by acetate extraction, Mn by phosphoric acid extraction and P by Olsen bicarbonate. The soil pH was quantified in a 1:1 (W/V) suspension of soil in distilled water. The total elements were measured by Inductively Coupled Plasma Mass Spectrometry (ICP-MS), except for the total Si element, which was performed with acetic acid extraction. See Pansu and Gautheyrou [47] for descriptions of the analysis methods.

### DNA extraction, amplification and sequencing

Each composite sample representing a plot was divided into 8 subsamples. Prior to DNA extraction, soil subsamples were ground for 30 minutes at 365 rpm with a Retsch PM 100 (Retsch GmbH, Haan, Germany). For all subsamples, the total DNA was extracted from 0.25g of soil using the PowerSoil DNA Isolation kit (MOBIO Laboratories, Carlsbad, CA, USA) following the manufacturer recommendations. Thereafter, the extracted DNA were pooled together to obtain a composite sample of DNA representing each plot. Verification of the DNA quality was performed by gel electrophoresis in a 0.8% agarose gel stained with ethidium bromide.

The V4 region of the bacterial 16S RNA gene was amplified using primers 515f (5’-GTGCCAGCMGCCGCGGTAA-3’)[48] and 806r (5’-GGACTACHVGGGTWTCTAAT-3’) [48] for the bacterial community, and the ITS2 region of the nuclear ribosomal RNA gene for the fungal community was amplified using primers fITS7 (5’-GTGARTCATCGAATCTTTG-3’) [49] and ITS4 (5’-TCCTCCGCTTATTGATATGC-3’) [50]. Eight independent PCR assays were performed for each DNA sample under the following conditions: 1 μL of template DNA, 2.5 μL of each primer (10 μM), and 25 μL of Master Mix Phusion GC buffer (New England Biolabs, Canada) for a final volume of 50 μL. Amplification was run on a Veriti Fast Thermal Cycler (Applied Biosystems) under the following conditions: 98°C for 30 s, 27 cycles of 10 s at 98°C, 60°C for 30 s and 72°C for 45 s, followed by 7 min at 72°C. An equimolar mix was created with the eight PCR products to obtain a composite PCR sample with a final concentration of 5 ng/μL for each sample. Samples were then purified with Clean PCR (Clean NA, GC Biotech BV, Netherlands) and paired end 2 x 250 bp length fragments were sequenced by Illumina (MiSeq), following Illumina recommendations. The Plateforme Génome-Transcriptome of the Université de Bordeaux conducted the PCR amplifications and Illumina sequencing.

### Illumina data processing

The Illumina sequence reads were processed using Mothur version 1.34.0 [51]. The forward and reverse sequences were aligned to generate ‘contigs’ for each sample. Sequences that contained one or more ambiguous reads and homopolymers greater than 8 bp were removed. Reads shorter than 250 bp and longer than 300 bp for bacteria, and shorter than 150 bp for fungi were discarded. Identical sequences were regrouped in unique sequences for shorter computational time. Then, putative chimeras were checked and removed using UCHIME [52], available on Mothur software. The number of sequences for each sample was normalised to have the same number as the smallest number of sequences found in a sample. Such an approach was undertaken to get unbiased estimators of the soil microbial diversity. Bacteria subsampling was performed for 3,025 sequences per sample. Compared to the bacteria, the fungal dataset had to be analysed twice. During the first analysis, all of the samples were normalised to 210 sequences, corresponding to the lowest number of reads found in our samples. The four Rivière Blanche sedge-dominated formations had the lowest number of sequences (between 210 and 300), which may be due to the low fungal richness explained by the particularly low plant cover in this plant formation (see Results and Discussion sections). The second time, to avoid an underestimation of fungal richness present in other plant formations, the four Rivière Blanche sedge-dominated formation samples were removed, and the remaining dataset was subsampled at 1,591 sequences per sample. Once normalised, uncorrected pairwise distances between the DNA sequences were calculated; then, the sequences were clustered to assign sequences to OTUs with 97% homology as commonly admitted [53–55] using the furthest neighbour method [56]. Each sequence, then each OTU, was taxonomically assigned by using the database UNITE [57] for fungi and SILVA v4 for bacteria [58]. Once all steps were done, all OTU sequences obtained in the first analysis (210 sequences subsampling) for Rivière Blanche sedge-dominated formations were compared one by one with the OTU sequences from the second dataset (1,591 sequences subsampling) and were merged to create the final dataset. A total of 798,073 reads for bacteria and 288,957 reads for fungi were obtained after the first sequence-processing step (S1 Table). After filtering, the numbers of remaining sequences fell to 322,807 reads for bacteria and 245,137 reads for fungi. Finally, 12,493 OTUs for bacteria and 3,741 OTUs for fungi were identified. After the exclusion of singletons, 6,539 OTUS for bacteria and 2,722 OTUs for fungi were used for further analyses. All data-trimming steps were conducted with Mothur software version 1.34.0. OTU tables reporting the taxonomic affiliation and the number of reads in each sample for each bacterial and fungal OTU are available in S2 Table. The Illumina MiSeq sequences are available under the NCBI SRA accession numbers SAMN05786746-SAMN05786777.

### Data analysis

Species richness (*S*), which correspond to the number of OTUs, Chao index [59], Simpson index (*1-D*) [60] and Pielou Evenness Index (*J*) [61] were calculated for each sample and by plant formation (*i*.*e*., by grouping the plots from the same vegetation type). The diversity indices were compared with a two-way Analysis of Variance (ANOVA) with site (*i*.*e*., Rivière Blanche and Kopéto sites) and plant formation (*i*.*e*., sedges dominated maquis, *Tristaniopsis spp*. maquis, *N*. *aequilateralis* monodominant rainforest and mixed rainforest) as fixed factors. Thereafter, *post-hoc* pairwise comparisons were performed using Tukey HSD tests. To observe whether there were significant differences in Ascomycota and Basidiomycota phyla abundances (based on the number of sequences) between plant formation at the two sites, Tukey HSD tests were also conducted among all pairwise comparisons after two-way ANOVA analyses with site and plant formation as factors. The OTUs defined for bacteria and fungi were used to perform rarefaction curves and rank-abundance diagrams. A Bray-Curtis dissimilarity index was calculated and ordinated by Nonmetric Multi-Dimensional Scaling (NMDS) [62] to observe the dissimilarities in microbial community composition per plot. Congruence of NMDS was tested by two-way PERMANOVA analyses [63], with again site and plant formation as factors. The Edaphic parameters per plot were analysed with a Principal Component Analysis (PCA) [64]. The relationships between bacterial and fungal communities were studied by plotting the Bray-Curtis distance matrices obtained for the bacterial and fungal datasets, and drawing a trending curve. Finally, to determine the relationships between bacterial or fungal community and environmental parameters (botanical inventory and edaphic parameter), distance-based linear model (distLM) were performed with PRIMER-E v7 software [65]. The distLM results were visualised with distance-based Redundancy Analysis (dbRDA) plots [66]. Mantel tests between bacteria and fungi distance matrix and each environmental factors distance matrix were also run for each site independently. All of the analyses were performed using R 3.1.1 (R Core Team 2014) and the ‘*vegan*’ R package, except for the distLM analyses.

## Results

### Bacterial and fungal composition and diversity

The taxonomic assignment for most sequences and OTUs found in this study indicated that members of Proteobacteria, Planctomycetes and Acidobacteria phyla were the most predominant in our bacterial dataset, followed by Actinobacteria and Verrucomicrobia (Table 1). Among the fungi, Basidiomycota and Ascomycota were the most dominant phyla, with other phyla representing less than 10% of all sequences and OTUs (*e*.*g*., Zygomycota, Glomeromycota, Chytridiomycota) (Table 1).

**Table 1:** Number of OTU and related percentages for the most abundant bacterial and fungal phyla, including for the latest *incertae sedis* phylum and unclassified fungi.

Bacteria	Nb. seq.	Nb. OTUs	Fungi	Nb. seq.	Nb. OTUs
Proteobacteria	3,920 (31.38%)	2,013 (30.78%)	Basidiomycota	1,534 (40.94%)	1,119 (41.11%)
Planctomycetes	2,352 (18.83%)	1,315 (20.11%)	Ascomycota	1,391 (37.12%)	953 (35.01%)
Acidobacteria	1,503 (12.03%)	930 (14.22%)	Zygomycota	240 (6.41%)	169 (6.21%)
Actinobacteria	1103 (8.83%)	666 (10.19%)	Glomeromycota	78 (2.08%)	52 (1.91%)
Verrucomicrobia	867 (6.94%)	527 (8.06%)	Chytridiomycota	59 (1.57%)	35 (1.29%)
Firmicutes	871 (6.97%)	338 (5.17%)	Rozellomycota	31 (0.83%)	19 (0.70%)
Chloroflexi	700 (5.60%)	325 (4.97%)	Fungi phylum *Incertae sedis*	11 (0.29%)	1 (0.04%)
Bacteroidetes	294 (2.35%)	131 (2.00%)	Unclassified fungi	171 (4.56%)	118 (4.34%)

**Fig 2:**
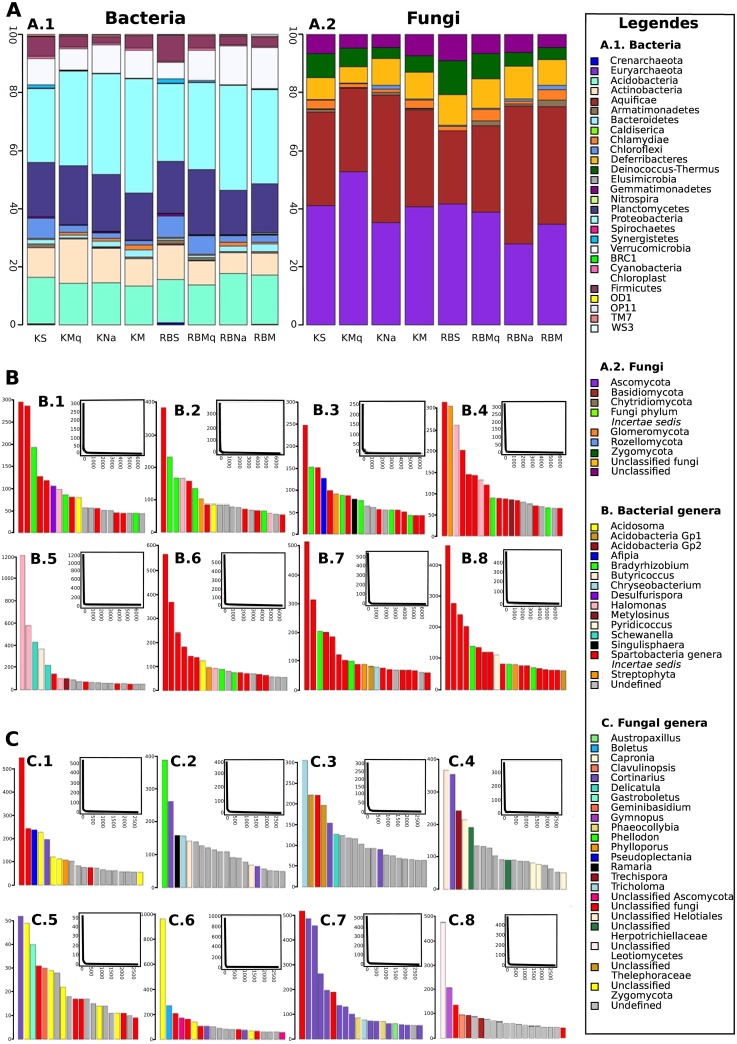
Phylum and genera composition of bacterial and fungal communities. **(A) Percentage of (A.1) bacterial and (A.2) fungal phyla in each plant formation at each site. Rank-abundance diagrams of the twenty most abundant (B) bacterial and (C) fungal OTUs, with the five most abundant genera identified for each formation at both site. The rank-abundances with all OTUs are presented at the upper right sides**. B.1 and C.1: Kopéto sedge-dominated formation (KS); B.2 and C.2: Kopéto *Tristaniopsis* spp. maquis (KMq); B.3 and C.3: Kopéto *N*. *aequilateralis* monodominant rainforest (KNa); B.4 and C.4: Kopéto mixed rainforest (KM); B.5 and C.5: Rivière Blanche sedge-dominated formation (RBS); B.6 and C.6: Rivière Blanche *Tristaniopsis* spp. maquis (RBMq); B.7 and C.7: Rivière Blanche *N*. *aequilateralis* monodominant rainforest (RBNa); and B.8 and C.8: Rivière Blanche mixed rainforest (RBM).

Based on the number of sequences per phylum, the phylum composition for each plant formation per site revealed that the most predominant bacterial phyla were the same regardless of the plant formation or site (Fig 2A.1). For fungi, the barplot per plant formation and site seems to indicate that Basidiomycota was the most abundant phylum in the two type of rainforest in Rivière Blanche and in *Nothofagus* dominant rainforest in Kopéto, whereas Ascomycota was the most abundant in all other plant formations (Fig 2A.2). ANOVA analyses, showing a significant interaction of the two plant formation and site variables (*F* = 6.752, *P* = 0.002 for Ascomycota and *F* = 12.934, *P* < 0.001 for Basidiomycota), and the following *post-hoc* Tukey HSD tests (S4 Fig), confirm these observations. The ratio of Ascomycota:Basidiomycota (A:B) was higher than 1 for the sedge dominated formations, *Tristaniopsis* spp. maquis and Kopéto mixed rainforest, and it was lower than 1 for the *N*. *aequilateralis* rainforests and the Rivière Blanche mixed rainforest (S4 Fig).

Rank-abundance diagrams obtained from bacterial and fungal OTUs indicated that for each plant formation and site, only a few numbers of OTUs were abundant (Fig 2B and 2C). For bacteria, one genus was predominantly present in each plant formation: *Spartobacteria genera Incertae sedis* (Fig 2B). However, for fungi no genus dominated in all plant formations and at all sites (Fig 2C).

Rarefaction curves were performed for each site on the bacterial and fungal datasets. The bacterial and fungal curves did not reach a plateau (S5 Fig), but the fungal curves had lower slopes than the bacterial curves and seemed to start to level off. However, the fact that the curves did not reach a plateau clearly indicates that the species diversity has not yet been assessed.

To assess the level of diversity within the bacterial and fungal communities, species richness (*S*) (here, the number of OTUs), Chao index, Simpson index (*1-D*) and Pielou evenness (*J*) were calculated by plots (S3 Table) and by plant formation per site (Table 2). ANOVA analyses revealed significant differences in species richness in both bacterial and fungal communities among the formations of the two sites (Table 3). Furthermore, we observed for the fungi the lowest species richness within the Rivière Blanche sedge-dominated formation (Table 2 and S3 Table). This low species richness at Rivière Blanche in sedge-dominated vegetation could be explained by the limited plant coverage.

**Table 2:** Diversity indices: species richness (*S*), Chao index, Simpson index (*1-D*) and Pielou evenness (*J*) for bacteria and fungi estimated per plant formation at the two study sites. Standard errors are indicated between brackets. The letters indicate significant differences between all pairwise comparisons determined using Tukey HSD tests (*P* < 0.05).

Microorganism type	Plant formation	Site	*S* (± SE)	*Chao* (± SE)	*1-D* (± SE)	*J* (± SE)
**Bacteria**	**Sedge-dominated formation**	**Kopéto**	1135 (± 151) a	4376 (± 107) ab	0.996 (± 0.002) a	0.912 (± 0.014) ab
		**Rivière Blanche**	839 (± 159) b	2654 (± 71) a	0.974 (± 0.033) a	0.827 (± 0.097) b
	***Tristaniopsis* spp maquis**	**Kopéto**	1163 (± 23) a	4305 (± 106) b	0.996 (± 0.001) a	0.907 (± 0.006) ab
		**Rivière Blanche**	1087 (± 188) ab	3744 (± 87) ab	0.991 (± 0.007) a	0.884 (± 0.047) ab
	***Nothofagus* rainforest**	**Kopéto**	1213 (± 51) a	4681 (± 114) b	0.997 (± 0.001) a	0.923 (± 0.012) a
		**Rivière Blanche**	1050 (± 48) ab	3953 (± 109) ab	0.993 (± 0.001) a	0.886 (± 0.006) ab
	**Mixed rainforest**	**Kopéto**	1008 (± 130) ab	3681 (± 92) ab	0.994 (± 0.003) a	0.887 (± 0.029) ab
		**Rivière Blanche**	1079 (± 31) ab	4086 (± 103) ab	0.994 (± 0.001) a	0.894 (± 0.009) ab
**Fungi**	**Sedge-dominated formation**	**Kopéto**	212 (± 58) a	1366 (± 102) a	0.952 (± 0.036) a	0.774 (± 0.071) ab
		**Rivière Blanche**	62 (± 13) b	310 (± 36) b	0.949 (± 0.026) a	0.870 (± 0.056) a
	***Tristaniopsis* spp maquis**	**Kopéto**	288 (± 39) ac	1114 (± 48) c	0.960 (± 0.033) a	0.798 (± 0.0.76) ab
		**Rivière Blanche**	225 (± 27) a	967 (± 58) ac	0.944 (± 0.045) a	0.772 (± 0.058) ab
	***Nothofagus* rainforest**	**Kopéto**	250 (± 23) ac	1439 (± 95) ac	0.964 (± 0.019) a	0.789 (± 0.056) ab
		**Rivière Blanche**	212 (± 29) a	1251 (± 92) ac	0.935 (± 0.025) a	0.725 (± 0.050) b
	**Mixed rainforest**	**Kopéto**	256 (± 42) ac	1374 (± 81) ac	0.964 (± 0.019) a	0.773 (± 0.061) ab
		**Rivière Blanche**	310 (± 30) c	1494 (± 103) ac	0.965 (± 0.029) a	0.824 (± 0.049) ab

**Table 3:** Two-way ANOVA table testing the effect of vegetation type, site, and their interaction on bacterial and fungal diversity indices (*S*: species richness, Chao index, *1-D*: Simpson index and *J*: Pielou evenness index).

	Bacteria			Fungi		
	*S*					
	df	*F*	*P*-value	df	*F*	*P*-value
**Formation**	3	2.899	0.056	3	26.319	**<0.001**
**Site**	1	8.121	0.009	1	15.842	**<0.001**
**Formation x Site**	3	3.553	0.029	3	11.533	**<0.001**
	**Chao**					
**Formation**	3	4.636	0.042	3	10.915	**0.003**
**Site**	1	2.744	0.065	1	17.900	**<0.001**
**Formation x Site**	3	3.116	0.045	3	7.907	**<0.001**
	***1-D***					
**Formation**	3	1.128	0.358	3	0.114	0.951
**Site**	1	2.896	0.102	1	0.596	0.448
**Formation x Site**	3	1.317	0.292	3	0.752	0.532
	***J***					
**Formation**	3	0.37	0.375	3	1.600	0.216
**Site**	1	0.02	0.023	1	0.440	0.514
**Formation x Site**	3	0.18	0.179	3	2.870	0.057

### Bacterial and fungal community structure

#### Floristic and site effects

**Fig 3:**
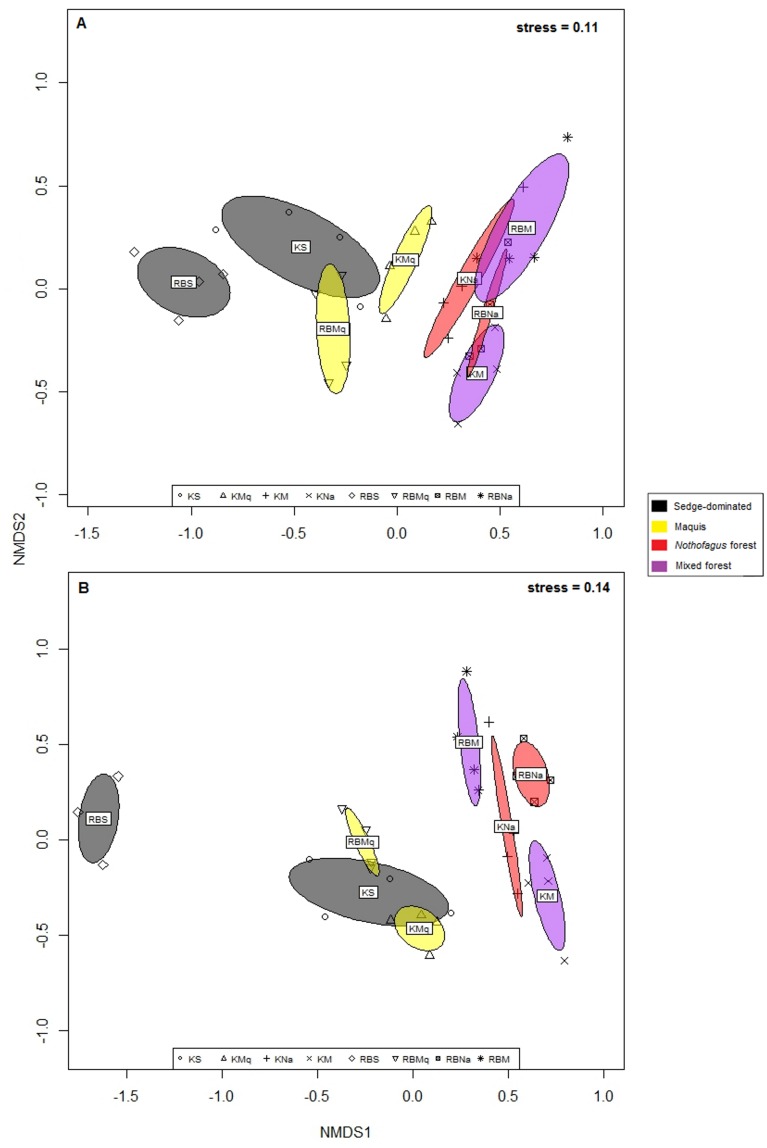
Nonmetric Multi-Dimensional Scaling (NMDS) ordination of Bray-Curtis dissimilarity for (A) the bacterial community and (B) the fungal community. K = Kopéto, RB = Rivière Blanche, S = sedge-dominated formation (in black), Mq = *Tristaniopsis* spp maquis (in yellow), Na = *N*. *aequilateralis* monodominant rainforest (in red), and M = mixed rainforest (in purple). The ellipses represent the 0.95 standard error calculated for each plant formation at each site.

To compare the OTU composition and abundance of the bacterial and fungal communities, NMDS ordinations, based on Bray-Curtis dissimilarity, were performed (Fig 3). The NMDS ordination indicated a distinction between forest and non-forest formations. Moreover, a distinction between monodominant and mixed rainforests was also visible. To corroborate the NMDS result, PERMANOVA were carried out (Table 4). The results indicated that the bacterial and fungal communities were significantly partitioned by the interaction between the two variables (plant formation x site) (Table 4). This interaction explained 15.8% and 14.8% of the variability of bacterial and fungal communities, respectively. PERMANOVA and NMDS were also conducted on the 210 sequences of fungal dataset and exhibited similar results to the 1,591 sequences dataset that were obtained (S6 Fig and S4 Table). Overall, the microbial communities were structured by the plant formation and site interaction.

**Table 4:** The effect of plant formation, site and their combination on the structure of bacterial and fungal communities assessed by two-way PERMANOVA.

Community	Factors	df	Sums of Sqs	MeanSqs	F.Model	Variation	Pr (>F)
**Bacteria**	**Formation**	3	2.3521	0.78404	4.6087	0.28461	**<0.001**
	**Site**	1	0.5196	0.51961	3.0544	0.06287	**<0.001**
	**Formation x Site**	3	1.3097	0.43655	2.5661	0.15847	**<0.001**
	**Residuals**	24	4.0829	0.17012	0.4940		
	**Total**	31	8.2643	1			
**Fungi**	**Formation**	3	2.5760	0.85867	2.5164	0.18991	**<0.001**
	**Site**	1	0.7785	0.77852	2.2815	0.05739	**<0.001**
	**Formation x Site**	3	2.0203	0.67344	1.9736	0.14894	**<0.001**
	**Residuals**	24	8.1896	0.34123	0.6038		
	**Total**	31	13.5645	1			

**Fig 4:**
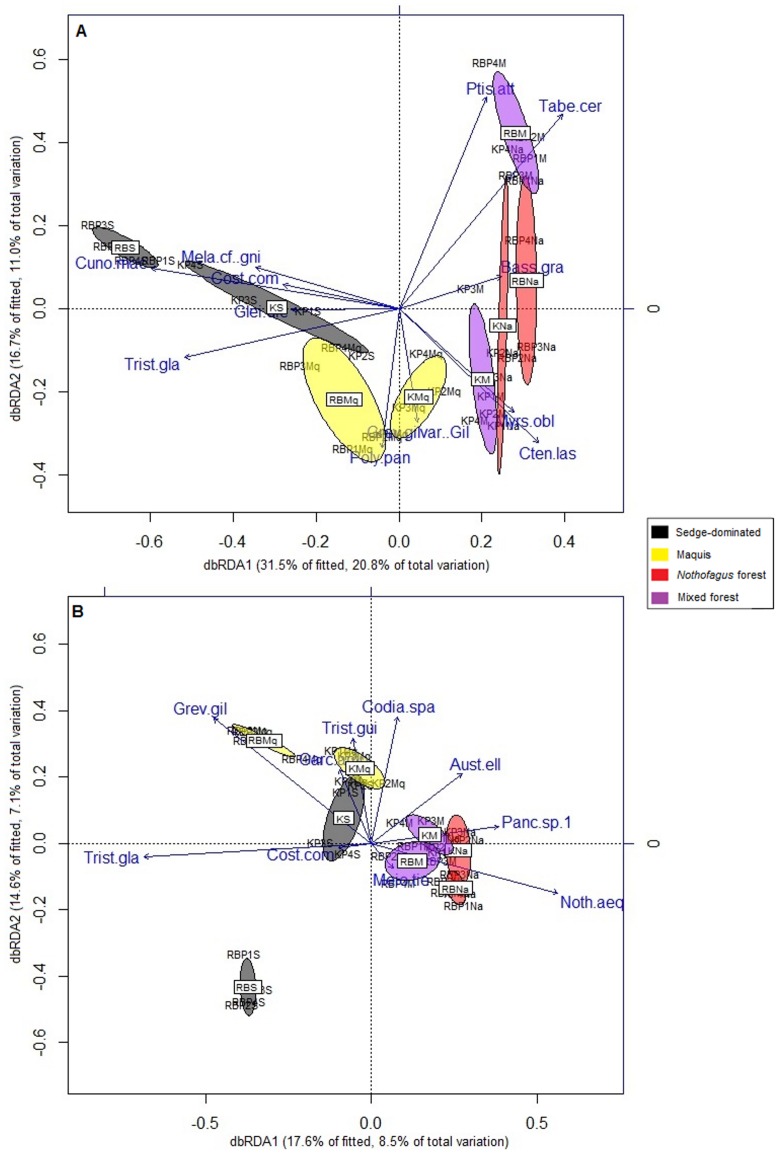
Distance-based Redundancy Analysis (dbRDA) ordination of the distance-based linear model (distLM) relating the (A) bacterial and (B) fungal communities to the floristic composition. Cuno.mac = *Cunonia macrophylla*, Tabe.cer = *Tabernaemontana cerifera*, Cten.las = *Ctenopteris lasiostipes*, Poly.pan = *Polyscias pancheri*, *Ptis*. *att = Ptisana attenuata*, Tris. gla = *Tristaniopsis glauca*, Grev. Gil.var.gil = *Grevillea gillivrayi var*. *gillivrayi*, Bass. Gra = *Basselinia gracilis* and Cost. com = *Costularia comosa*, Mela cf. gni = *Melaleuca cf*. *gnioides*, Glei dic = *Gleichenia dicarpa* and Myrs obl = *Myrsine oblanceolata*.

The distLM and the dbRDA ordination performed for bacteria showed that the bacterial communities (Fig 4A and S5 Table) were significantly related to the following plant species: *Cunonia macrophylla* (Cunoniaceae), *Tabernaemontana cerifera* (Apocynaceae), *Ctenopteris lasiostipes* (Polypodiaceae), *Polyscias pancheri* (Araliaceae), *Ptisana attenuata* (Marattiaceae), *Tristaniopsis glauca* (Myrtaceae), *Grevillea gillivrayi* var. *gillivray* (Proteaceae), *Basselinia gracilis* (Arecaceae), *Costularia comosa* (Cyperaceae), *Melaleuca cf*. *gnioides* (Myrtaceae), *Gleichenia dicarpa* (Gleicheniaceae) and *Myrsine oblanceolata* (Primulaceae) (S5 Table). This link between the bacterial community and floristic composition was confirmed with Mantel tests (Kopéto: r = 0.548, p = 0.001; Rivière Blanche: r = 0.6657, p = 0.001).

As with bacteria, the fungal community was also significantly related to several plant species (Fig 4B and S5 Table), namely the following: *Tristaniopsis glauca* (Myrtaceae), *Grevillea gillivrayi* (Proteaceae), *Codia spatulata* (Cunoniaceae), *Nothofagus aequilateralis* (Nothofagaceae), *Pancheria* sp1 (Cunoniaceae), *Costularia comosa* (Cyperaceae), *Meiogyne tiebaghiensis* (Annonaceae), *Tristaniopsis guillainii* (Myrtaceae), *Austrobuxus ellipticus* (Picrodendraceae) and *Garcinia amplexicaulis* (Clusiaceae). Like bacteria, the Mantel tests between fungi and floristic composition indicated a significant correlation between them (Kopéto: r = 0.6588, p = 0.001; Rivière Blanche: r = 0.6395, p = 0.001). Interestingly, only two species were found to be significantly related to both bacterial and fungal communities: *Costularia comosa* and *Tristaniopsis glauca*.

### Edaphic parameters

Prior to the distLM analyses, a Principal Component Analysis (PCA) was performed to determine which edaphic parameters were related to each plant formation (S7 Fig). The first axis explained 59.20% of the observed variance, and the second axis explained 15.46%. There was a clear distinction between non-forest (on the right side of the plots) and forest formations (on the left side of the plot) (S7 Fig). The parameter factor map shows that the parameters related to plant growth, such as nutrients (phosphorus (P), sodium (Na), magnesium (Mg) and potassium(K)), organic matter (OM) and total nitrogen (Total.N), are on the left side of the map, such as the rainforests (S7 Fig). To the opposite, on the right side of the map are grouped edaphic parameters which are usually associated with low soil fertility, a high content of sulphur (S) and total iron (Fe.tot), and a high potassium/magnesium ratio (ratio.K.Mg) (S7 Fig). A gradient seems to be observable from the sedge-dominated formation with low fertility soil to rainforests, where soil fertility parameters are high. In the middle of the map, *Tristaniopsis*-dominated maquis are plotted with average values for each edaphic parameter present in the PCA analysis (S7 Fig).

**Fig 5:**
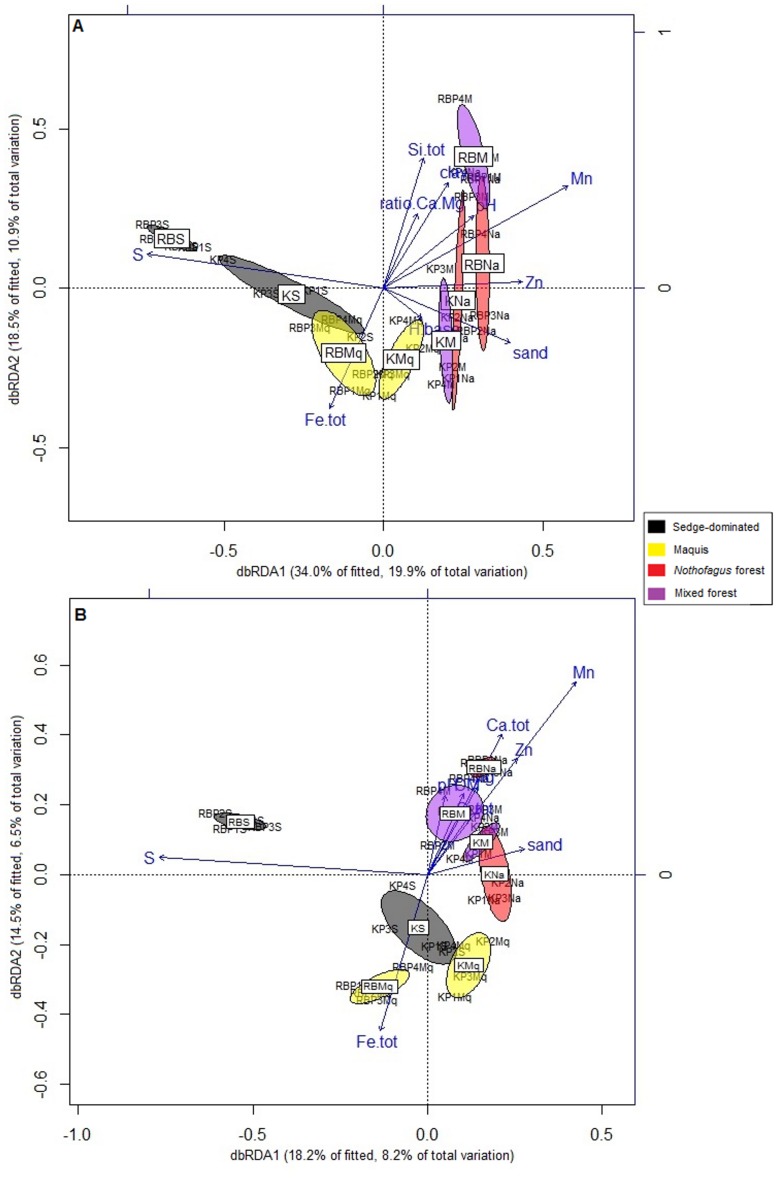
Distance-based Redundancy Analysis (dbRDA) of the (A) bacterial and (B) fungal communities and edaphic parameters.

The distLM analyses and the dbRDA ordinations conducted between the microbial communities and the edaphic parameters indicate significant relationships between bacterial and fungal community structures and different edaphic parameters (Fig 5 and S6 Table). The distLM that was performed on edaphic parameters shows a significant relationship between the bacterial community and exchangeable sulphur (S), the percentage of exchangeable Hydrogen (H.base), exchangeable Zinc (Zn), the percentage of clay (clay), total iron (Fe.tot), exchangeable Manganese (Mn), pH, total Silicium (Si.tot), Calcium/Magnesium ratio (Ca:Mg ratio) and the percentage of sand (sand) (S6 Table). Concerning fungi, the distLM analysis indicated that exchangeable sulphur (S), total iron (Fe.tot), total calcium (Ca.tot), exchangeable zinc (Zn), total silica (Si.tot), the percentage of sand (sand), pH, exchangeable magnesium (Mg), organic matter (OM) and exchangeable manganese (Mn) were significantly related to this soil microbial community (S6 Table). Overall, the bacterial and fungal communities are thus related to the soil fertility, such as the organic matter and the nutrient content. However, nitrogen (Total.N) and organic carbon (Organic.C), which are well-known to be correlated with fungi, were not found to be related to the fungal community here.

#### Relationships between the bacterial and fungal communities

**Fig 6:**
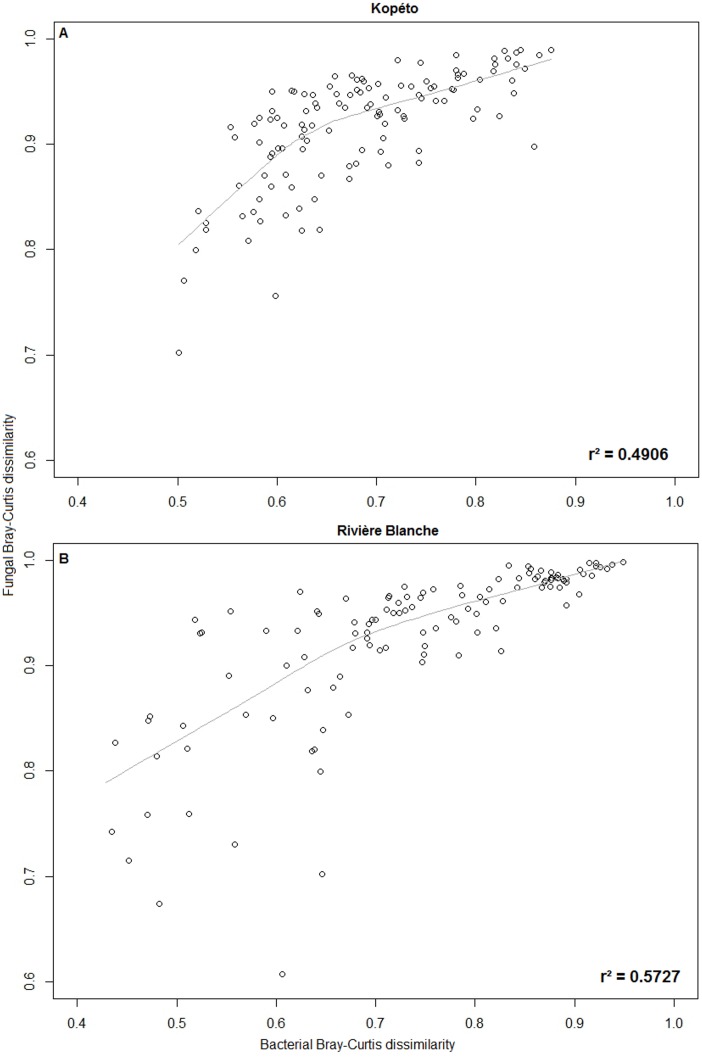
Scatter plots and trending curves between the bacterial and fungal communities at the (A) Kopéto site and (B) Rivière Blanche site.

The scatter plots of distance matrices of bacterial and fungal communities suggest a correlation between bacteria and fungi within each geographical location (R^2^ = 0.4906 for bacteria and R^2^ = 0.5727 for fungi) (Fig 6). This observation was supported by Mantel test results (r = 0.7004 and *p* = 0.001 for Kopéto; r = 0.7568 and *p* = 0.001 for Rivière Blanche).

## Discussion

### Effect of plant cover on soil microbial diversity

The different studied vegetation types present different levels of field openness. This discrepancy in plant cover seems to have an impact on soil microbial richness. Indeed, the sedge-dominated formation at the Rivière Blanche site showed the lowest fungal OTUs richness, and to a lower extent also the lowest bacterial OTUs richness. This formation was characterised by very low plant coverage (between 15 and 30%), contrary to the others, which were 70 to 95% covered. The difference in the soil microbial community due to plant cover was demonstrated by Knelman *et al*. [11], who found that the presence or absence of vegetation in a primary succession after glacier retreat has an impact on soil microbial community structure. A study conducted on unvegetated and forest soils on primary succession also revealed a difference in the microbial community [67].

NMDS and PERMANOVA analyses clearly indicated that bacterial and fungal communities were both structured by the type of plant formation. Thus, vegetation covers, in plus to affect the species richness as indicated above, impact species composition and their abundance. Furthermore, we also found that soil microorganism communities were related to the dominant plant species, such as *T*. *glauca* and *N*. *aequilateralis*. Such an effect of plant formation on microbial communities may be due to different factors, notably host preference, litter quality and root exudates [68,69]. *Tristaniopsis* and *Nothofagus* species are known to be involved in a mutualistic association with fungi, called ectomycorrhizal symbiosis [44]. Several studies have shown host preference in ectomycorrhizal fungal communities [70,71]. Bacteria can also present a host preference [72,73]. Indeed, nitrogen-fixing bacteria are associated with the roots of certain plant groups. For instance, *Rhizobium* is commonly associated with leguminous plants [72], or Actinomycetes such as *Frankia* with *Casuarina* plants [73]. In our dataset, ECM fungi and bacteria, such as *Bradyrhizobium* were present and may subsequently explain the plant formation structure observed in microbial communities. Plant species may also impact the soil microbial community *via* the type of litter produced [74,75]. Moreover, root exudates can affect the soil microorganisms. Each plant species produces root exudates (mainly carbohydrates, organic acids and amino acids), which have been determined to vary in terms of their composition from each other [76]. These exudates attract some particular microorganisms that can improve plant growth [4,77]. At the same time, roots from certain plant species can also produce exudates containing antimicrobial metabolites [76]. The effects of exudates on microbial communities are well studied in bacteria and have been shown to be more effective on these microorganisms than on fungi [76,78,79]. However, it had also been demonstrated that soil fungal communities are regulated by plant roots exudates [80]. In summary, the effect of plant formation and plant species on microbial communities may be explained by the narrow relationships between soil microorganisms and plant composition.

### Microbial diversity and perturbation effect along a chronosequence

Studies conducted on New Caledonian ultramafic substrates indicated that in the natural environment, the bacterial community was dominated by Proteobacteria and Acidobacteria [81,82]. In other geographic regions, Actinobacteria and/or Acidobacteria were observed as the dominant phyla [83–85]. In the present work, bacterial communities were dominated by Proteobacteria, Planctomycetes, Acidobacteria and Actinobacteria. The composition did not really differ from other studies [81–85], except for the Planctomycetes. Indeed, this phylum was highly present within all of the plant formations studied. To our knowledge, such an abundance of Planctomycetes in the soil has never been shown [86,87]. Buckley *et al*. [88] had observed a higher abundance of this phylum in soil that was cultivated 50 years ago compared to soil still actively managed. The Planctomycetes has been revealed to be sensitive to soil history [88,89]. Planctomycetes phylum could be an indicator of an ancient environmental disturbance.

Regarding fungal communities, a higher proportion of Ascomycota phylum than Basidiomycota phylum was detected in sedge-dominated formations, *Tristaniopsis*-dominated maquis and Kopéto mixed rainforest. Numerous studies have shown a pattern of greater representation of Ascomycota than Basidiomycota on managed lands, such as in pastures and vineyards [90–92]. However, within rainforests, Basidiomycota were detected as the most abundant phylum [93,94]. A larger representation of Ascomycota could be an indicator of ecosystem degradation. Indeed, sedge- and *Tristaniopsis*-dominated formations are known to result from past bushfire perturbations. Similarly, the mixed rainforest at the Kopéto site seems to correspond to a degraded rainforest. In summary, the presence of Ascomycota and Planctomycetes in higher abundance could represent biological indicators of past disturbances that occurred on different time scales.

### Relationship between soil microbial communities and edaphic parameters

The diverse statistical analyses performed (distLM, dbRDA ordinations and Mantel tests) indicated that the bacterial and fungal communities were related to several edaphic parameters. The most striking differences in soil properties were the higher content of iron, sulphur and potassium/magnesium ratio in sedge-dominated and *Tristaniopsis*-dominated formations as well as the higher content of nutrients and organic matter in the rainforests. The greater abundance of nutrients in rainforests may be simply explained by the larger litter production in these systems. Not mutually exclusive, this could also result from higher decomposition rates. Litter decomposition depends on the type of litter and the decomposers involved in the degradation process [95,96].

This difference in litter degradation, between a forest and non-forest formation, could also be due to solar radiation that reaches the ground and acts indirectly on soil microorganisms. Indeed, in the presence of scarce vegetation on the ground, solar radiation that interacts with soil was much higher than in rainforest soil, which has an effect on microbial activity that leads to a change in microbial litter composition [97]. This solar radiation decrease litter degradation and affect soil carbon cycling due to a loss of carbon released directly into atmosphere, rather than into the soil organic matter pool [98].

Ultramafic substrates are characterised by a high nickel concentration that may impact microbial composition by selecting microorganisms that are tolerant to this metal [99–101]. In New Caledonia, studies have demonstrated that the presence of AMF improves plant tolerance to nickel [102,103] and also the existence of nickel-tolerant ECM fungal *Pisolithus albus* isolates [36]. Furthermore, in these types of soils, certain bacterial strains known to be nickel-resistant and nickel-hyperaccumulators are present [99,100]. These nickel-hyperaccumulator bacteria have been previously found in New Caledonia soil [104]. The presence of these microorganisms may explain the relationship observed between edaphic parameters (such as nickel) and microbial community structure.

### Site effect on soil microbial diversity

A very recent study conducted at one site on a different isolated ultramafic Massif in New Caledonia, namely the ‘Koniambo Massif’, indicated an effect from the aboveground vegetation and edaphic parameters on microbial composition and abundance using a 454 pyrosequencing approach [82]. As previously indicated, the results obtained in the present study confirm such patterns. However, NMDS and PERMANOVA analyses clearly revealed a geographical structure of the bacterial and the fungal communities. In other words, each site harbours its own microbial community. In New Caledonia, there is a geographic discontinuity of ultramafic substrates, which may limit the dispersion of microorganisms. MacArthur and Wilson [105] argued with their biogeographical island theory that the richness of organisms decreases when the distance of the island from the continent increases, causing an isolation of the population. Peay *et al*. [106,107] revealed such a pattern in ectomycorrhizal fungi. Ultramafic substrates in New Caledonia are represented by two types: a large massif covering the South of the main island (‘Massif du Grand Sud’) and several isolated islets distributed primarily along the west coast. In some ways, this type of distribution may be compared to a continent *versus* islands system, where the 'Massif du Grand Sud' may be considered as the mainland, with the isolated islands more or less distant from this ‘continent’. Differences in soil microbial communities between sites may thus be explained by dispersal limitations in the context of the biogeographical island theory. To validate such a hypothesis, in the future, a new sampling approach must be undertaken by adding sites from the 'Massif du Grand Sud' and from other isolated ultramafic islets, using the same field and molecular procedures.

### Relationship between soil bacterial and fungal communities

We have previously observed that bacterial and fungal soil communities are related to plant cover, edaphic variables and geographical factors. However, these soil communities may be related to other parameters, such as the interplay between them. Indeed, mantel tests revealed a significant correlation between bacteria and fungi at both sites. Some bacteria are commonly found on fungal hyphae, spores, mycorrhizal roots, fruiting bodies and inside fungi [108,109]. For these bacteria, the fungi are required for their survival. Furthermore, fungi produce some exudates that are primary or exclusive nutrients sources for these bacteria [21]. These compounds seem to be used as a mechanism to select groups of bacteria associated with ECM fungi [19]. These groups of bacteria, called Mycorrhiza Helper Bacteria (MHB), were shown to improve fungal mycorhization [19], such as *Pseudomonas monteilii* which increased ECM and AM colonisation as well as induced the formation of rhizobial nodules on *Acacia holoserica* [20]. The effect of MHB was demonstrated to be fungal specific, not plant specific [20]. In our dataset, bacteria genera known to be MHB were detected, which may partially explain the relationship between bacteria and fungi.

## Conclusion

In the present work, a metabarcoding approach was used to assess bacterial and fungal diversity and the biotic and abiotic factors potentially shaping this diversity on ultramafic outcrops in New Caledonia. Four distinct plant formations of a potential chronosequence on two distinct sites located in the South and North of the main island were studied. Microbial richness, composition and abundance were linked to the plant cover type and especially to the dominant plant species, namely *N*. *aequilateralis* and *T*. *glauca*. Interestingly, a larger proportion of Ascomycota was observed in non-rainforest formations and within the mixed rainforest at the Kopéto site, which may be an indicator of ecosystem degradation. Bacterial phyla composition showed a very high proportion of Planctomycetes. To our knowledge, such a pattern has never been observed [86,82] and could be, as for Ascomycota, an indicator of past disturbances, but that occurred there much longer ago. Furthermore, soil bacterial and fungal communities were influenced by diverse edaphic parameters as well as by the interplay between them. Another very interesting finding is the existence of a site effect. Differences in microbial communities between geographical locations may be explained by dispersal limitation in the context of the biogeographical island theory of MacArthur and Wilson [105]. Finally, each plant formation at each site possesses is own microbial community resulting from complex interactions between abiotic and biotic factors. Such observations have a direct implication for conservation purposes. Indeed, they support the fact that efforts toward protecting the remaining rainforest fragments must be strongly maintained.

## Supporting Information

S1 Fig Nonmetric Multi-Dimensional Scaling (NMDS) plot based on pairwise Bray-Curtis dissimilarities between plant communities. Sites: K = Kopéto, RB = Rivière Blanche; and Plant formations: S = Sedge-dominated formation, Mq = *Tristaniopsis* spp maquis, Na = *N*. *aequilateralis* monodominant rainforest, M = Mixed rainforest. The ellipses represent the 0.95 standard error limits for each plant formation per site.
(TIF)

S2 Fig Orthophotos representing the repartition of the 20m x 20m plots at the (A) Kopéto site and (B) Rivière Blanche site. Each plant formation is represented by a colour: sedge-dominated formation in black, *Tristaniopsis* spp. maquis in yellow, *N*. *aequilateralis* monodominant rainforest in red and mixed rainforest in purple. For each plot, the names are related to the study site: K = Kopéto, RB = Rivière Blanche; the number of the plots in the same plant formation: P1-P4 = Plot 1 to 4; and the plant formation: S = Sedge-dominated formation, Mq = *Tristaniopsis* spp. maquis, Na = *N*. *aequilateralis* monodominant rainforest, and M = mixed rainforest.
(TIF)

S3 Fig Scheme representing the 20 m by 20 m grid installed in each plot. A soil core was collected from each sampling point (red dots), and these were grouped together to create a composite sample per plot.
(TIF)

S4 Fig The mean abundance of Ascomycota and Basidiomycota phyla in each plant formation at the two study sites. The bars represent the standard error. Different letters, a lowercase letter for Ascomycota and an uppercase letter for Basidiomycota, indicate significant differences among plant formations and sites, as determined by Tukey HSD tests (*P*<0.05). The values of Ascomycota:Basidiomycota (A:B) ratios are indicated.
(TIF)

S5 Fig Rarefaction curves obtained from OTU distribution for (A and B) bacterial and (C and D) fungal datasets at (A and C) Kopéto and (B and D) Rivière Blanche sites. Sedge-dominated vegetation is represented in black, *Tristaniopsis* spp. maquis in yellow, *N*. *aequilateralis* monodominant rainforest in red and mixed rainforest in purple.
(TIF)

S6 Fig Nonmetric Multi-Dimensional Scaling (NMDS) ordination of Bray-Curtis dissimilarities between the fungal communities in soil using the 210 sequences dataset. Plant formation names: K = Kopéto, RB = Rivière Blanche, S = sedge-dominated formation, Mq = *Tristaniopsis* spp maquis, Na = *N*. *aequilateralis* monodominant rainforest, and M = mixed rainforest. The ellipses represent the 0.95 standard error limit for each plant formation at each site.
(TIF)

S7 Fig Principal Component Analysis performed from the edaphic parameters of each plot. (TIF)

S1 Table Number of sequences remaining after each analysis step for the bacterial and fungal datasets. (PDF)

S2 Table OTU tables presenting the taxonomic assignation and the number of reads in each sample for each bacterial and fungal OTU. (XLSX)

S3 Table Diversity indices: species richness (*S*), Chao index, Simpson index (*1-D*) and Pielou evenness (*J*) for bacteria and fungi estimated per plot. (PDF)

S4 Table The effect of plant formation, site and their combination on the structure of the 210 sequences in the fungal community dataset assessed by PERMANOVA. (PDF)

S5 Table Summary of the distance-based linear model (distLM) results for the floristic variables that were significantly related to the bacterial and fungal communities. The R^2^ that corresponds to the proportion of explained variation in the model by sequentially adding environmental variables, the proportion of variance explained for each variable, the sum of squares (SS), the pseudo-*F* statistic (analogous to Fisher’s *F* test), and the *P*-value are presented.
(PDF)

S6 Table Summary of the distance-based linear model (distLM) results for the edaphic variables that were significantly related to the bacterial and fungal communities. The R^2^ that corresponds to the proportion of explained variation in the model by sequentially adding environmental variables, the proportion of variance explained for each variable, the sum of squares (SS), the pseudo-*F* statistic (analogous to Fisher’s *F* test) and the *P*-value are presented.
(PDF)
