# Supplementary figures and images for: Environmental and Geographical Factors Structure Soil Microbial Diversity in New Caledonian Ultramafic Substrates: A Metagenomic Approach
Source: PLoS One. 2016 Dec 1;11(12):e0167405. doi: 10.1371/journal.pone.0167405 (PMC5131939; doi:10.1371/journal.pone.0167405)

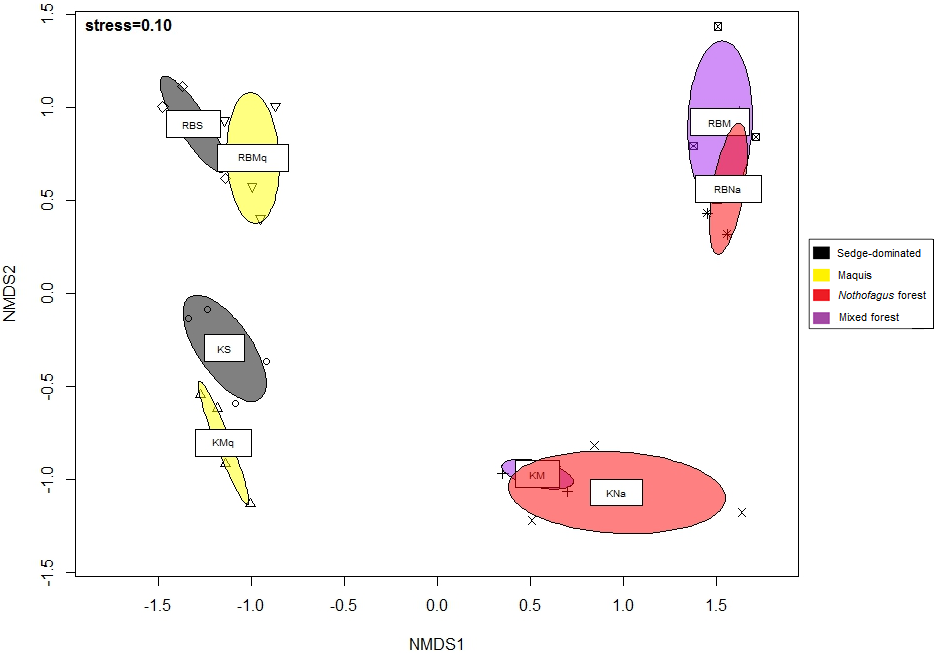

Supplement: S1 Fig — Sites: K = Kopéto, RB = Rivière Blanche; and Plant formations: S = Sedge-dominated formation, Mq = Tristaniopsis spp maquis, Na = N. aequilateralis monodominant rainforest, M = Mixed rainforest. The ellipses represent the 0.95 standard error limits for each plant formation per site. (TIF) [file pone.0167405.s001.tif]

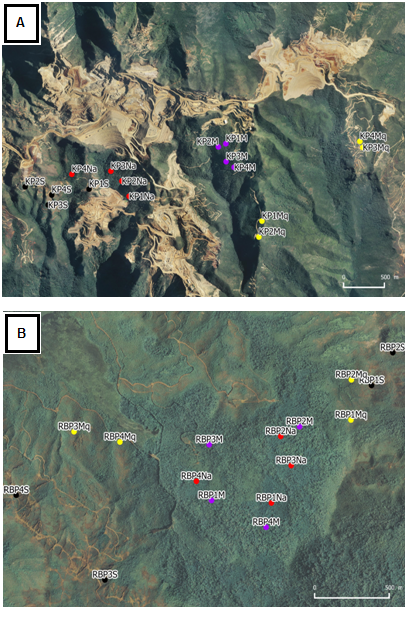

Supplement: S2 Fig — Each plant formation is represented by a colour: sedge-dominated formation in black, Tristaniopsis spp. maquis in yellow, N. aequilateralis monodominant rainforest in red and mixed rainforest in purple. For each plot, the names are related to the study site: K = Kopéto, RB = Rivière Blanche; the number of the plots in the same plant formation: P1-P4 = Plot 1 to 4; and the plant formation: S = Sedge-dominated formation, Mq = Tristaniopsis spp. maquis, Na = N. aequilateralis monodominant rainforest, and M = mixed rainforest. (TIF) [file pone.0167405.s002.tif]

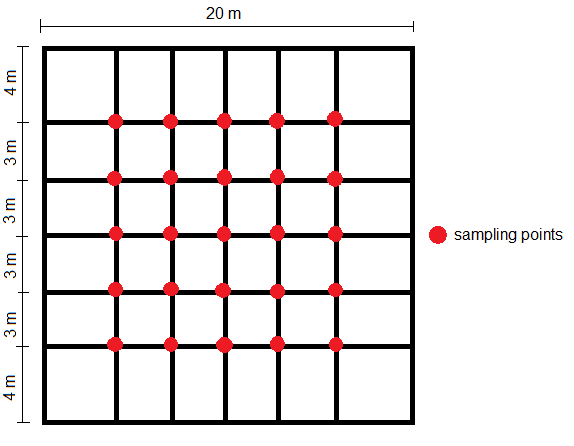

Supplement: S3 Fig — A soil core was collected from each sampling point (red dots), and these were grouped together to create a composite sample per plot. (TIF) [file pone.0167405.s003.tif]

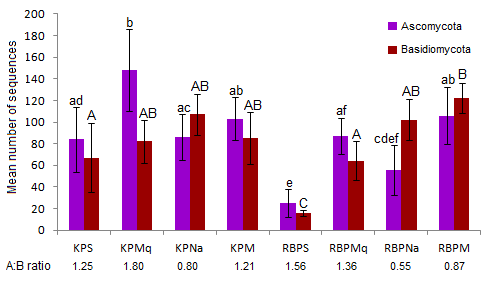

Supplement: S4 Fig — The bars represent the standard error. Different letters, a lowercase letter for Ascomycota and an uppercase letter for Basidiomycota, indicate significant differences among plant formations and sites, as determined by Tukey HSD tests (P<0.05). The values of Ascomycota:Basidiomycota (A:B) ratios are indicated. (TIF) [file pone.0167405.s004.tif]

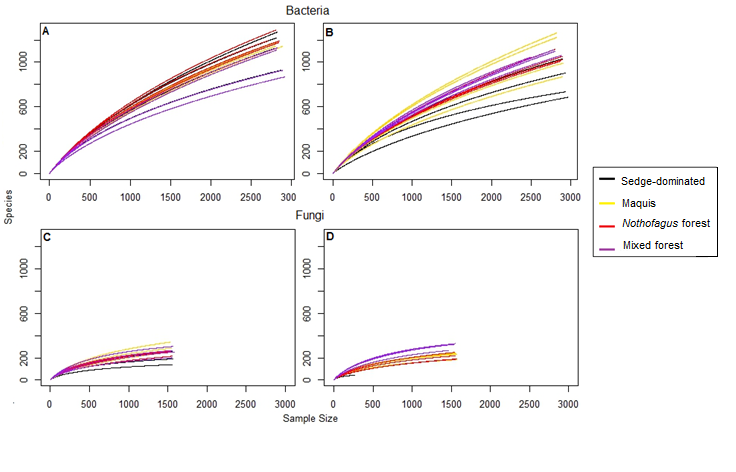

Supplement: S5 Fig — Sedge-dominated vegetation is represented in black, Tristaniopsis spp. maquis in yellow, N. aequilateralis monodominant rainforest in red and mixed rainforest in purple. (TIF) [file pone.0167405.s005.tif]

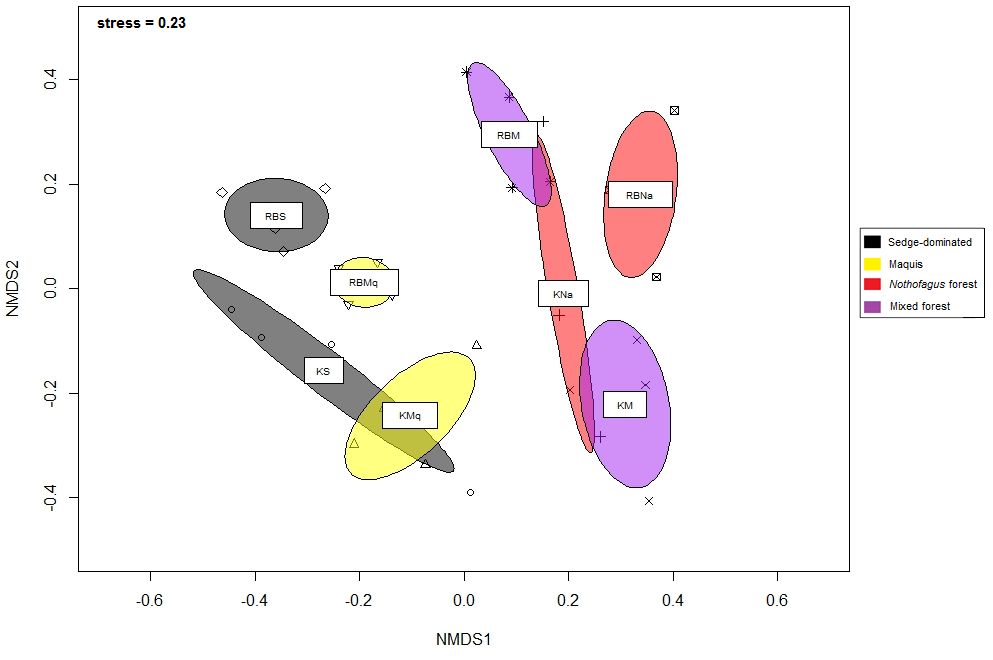

Supplement: S6 Fig — Plant formation names: K = Kopéto, RB = Rivière Blanche, S = sedge-dominated formation, Mq = Tristaniopsis spp maquis, Na = N. aequilateralis monodominant rainforest, and M = mixed rainforest. The ellipses represent the 0.95 standard error limit for each plant formation at each site. (TIF) [file pone.0167405.s006.tif]

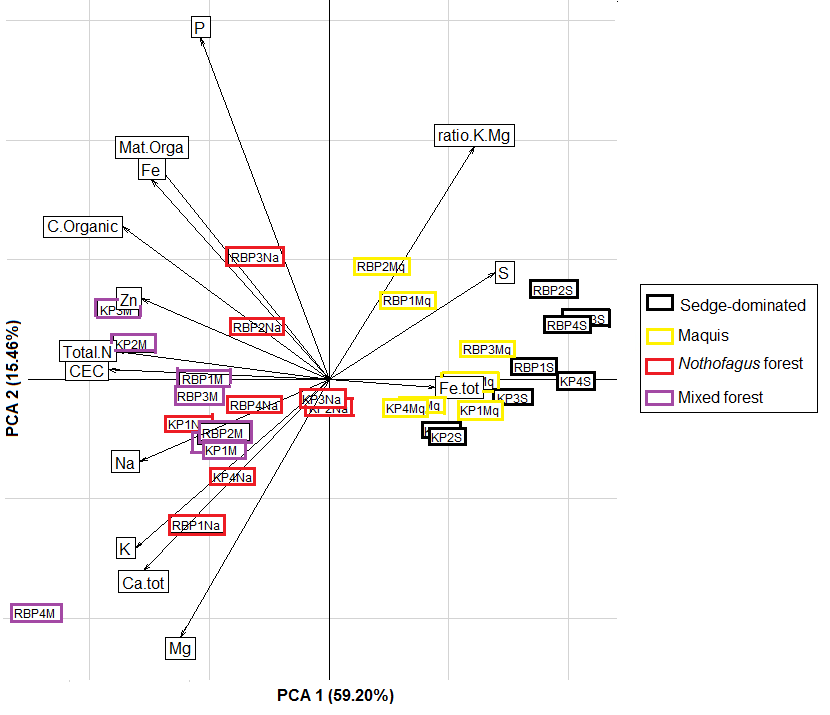

Supplement: S7 Fig — (TIF) [file pone.0167405.s007.tif]
