# Supplementary material for: Environmental and Geographical Factors Structure Soil Microbial Diversity in New Caledonian Ultramafic Substrates: A Metagenomic Approach
Source: PLoS One. 2016 Dec 1;11(12):e0167405. doi: 10.1371/journal.pone.0167405 (PMC5131939; doi:10.1371/journal.pone.0167405)
Supplement: S1 Table — (PDF) [file pone.0167405.s008.pdf]

| | Bacteria | Fungi |
|---|---|---|
| raw sequence | 798,073 | 288,957 |
| filtered sequence | 322,807 | 245,137 |
| pre-cluster sequence | 162,812 | -* |
| chimeric sequence | 112,801 | 47,102 |
| subsampling sequence | 96,800 | 6,720**/44,548*** |
| nb OTU | 12,493 | 1,480**/3,741*** |

* This step is not realised for fungal dataset

** Number of sequences obtained for the analysis with subsampling at 210 sequences per sample

*** Number of sequences obtained for the analysis whith subsampling at 1,591 sequences per sample
