# Supplementary material for: Environmental and Geographical Factors Structure Soil Microbial Diversity in New Caledonian Ultramafic Substrates: A Metagenomic Approach
Source: PLoS One. 2016 Dec 1;11(12):e0167405. doi: 10.1371/journal.pone.0167405 (PMC5131939; doi:10.1371/journal.pone.0167405)
Supplement: S3 Table — (PDF) [file pone.0167405.s010.pdf]

Site	plot	Bacteria			Fungi		
		S	1-D	J	S	1-D	J
Kopéto	KP1S	1,269	0.997	0.923	260	0.981	0.845
	KP2S	1,219	0.997	0.925	259	0.968	0.797
	KP3S	1,123	0.994	0.898	190	0.957	0.779
	KP4S	928	0.995	0.9	140	0.9	0.676
	KP1Mq	1,141	0.995	0.899	342	0.964	0.821
	KP2Mq	1,146	0.996	0.91	268	0.979	0.832
	KP3Mq	1,185	0.995	0.907	288	0.985	0.852
	KP4Mq	1,181	0.996	0.913	253	0.913	0.685
	KP1Na	1,175	0.995	0.907	216	0.949	0.735
	KP2Na	1,193	0.997	0.919	257	0.985	0.858
	KP3Na	1,289	0.997	0.933	265	0.947	0.752
	KP4Na	1,196	0.997	0.931	261	0.974	0.812
	KP1M	1,129	0.997	0.917	268	0.963	0.799
	KP2M	1,108	0.994	0.891	303	0.979	0.846
	KP3M	868	0.989	0.848	253	0.931	0.72
	KP4M	927	0.994	0.893	201	0.928	0.725
Rivière Blanche	RBP1S	686	0.924	0.683	59	0.947	0.86
	RBP2S	904	0.988	0.863	70	0.971	0.92
	RBP3S	734	0.99	0.869	44	0.914	0.795
	RBP4S	1,031	0.994	0.893	73	0.965	0.903
	RBP1Mq	869	0.982	0.826	184	0.877	0.688
	RBP2Mq	992	0.989	0.865	231	0.969	0.812
	RBP3Mq	1,223	0.996	0.919	243	0.969	0.809
	RBP4Mq	1,263	0.997	0.926	240	0.959	0.777
	RBP1Na	1,116	0.994	0.894	219	0.948	0.75
	RBP2Na	1,024	0.992	0.884	189	0.932	0.714
	RBP3Na	1,008	0.993	0.883	250	0.959	0.776
	RBP4Na	1,053	0.993	0.881	191	0.901	0.661
	RBP1M	1,099	0.994	0.889	321	0.974	0.845
	RBP2M	1,110	0.995	0.897	325	0.982	0.854
	RBP3M	1,060	0.993	0.884	329	0.981	0.846
	RBP4M	1,045	0.994	0.905	266	0.922	0.75
