# Supplementary material for: Environmental and Geographical Factors Structure Soil Microbial Diversity in New Caledonian Ultramafic Substrates: A Metagenomic Approach
Source: PLoS One. 2016 Dec 1;11(12):e0167405. doi: 10.1371/journal.pone.0167405 (PMC5131939; doi:10.1371/journal.pone.0167405)
Supplement: S4 Table — (PDF) [file pone.0167405.s011.pdf]

Permanova result	Df	SumsOfSqs	MeanSqs	F.Model	Variation	Pr(>F)
Formation	3	2.2449	0.74828	2.0348	0.16584	0.001
Site	1	0.7732	0.77323	2.1026	0.05712	0.001
Formation x site	3	1.6919	0.56396	1.5336	0.12499	0.001
Residuals	24	8.8259	0.36775	0.65204		
Total	31	13.5359	1			
