# Supplementary material for: Environmental and Geographical Factors Structure Soil Microbial Diversity in New Caledonian Ultramafic Substrates: A Metagenomic Approach
Source: PLoS One. 2016 Dec 1;11(12):e0167405. doi: 10.1371/journal.pone.0167405 (PMC5131939; doi:10.1371/journal.pone.0167405)
Supplement: S5 Table — The R2 that corresponds to the proportion of explained variation in the model by sequentially adding environmental variables, the proportion of variance explained for each variable, the sum of squares (SS), the pseudo-F statistic (analogous to Fisher’s F test), and the P-value are presented. (PDF) [file pone.0167405.s012.pdf]

	Variable	Abbreviation	R^2	% variance explained	SS (trace)	Pseudo-*F*	*P* -value
**Bacteria**	*Cunonia macrophylla*	Cuno mac	0.13120	0.13120	10843.0	4.5303	0.001
	Tabernaemontana cerifera	Tabe cer	0.21565	0.08446	6979.7	3.1226	0.001
	Ctenopteris lasiostipes	Cten las	0.29032	0.07467	6171.0	2.9461	0.001
	Polyscias pancheri	Poly pan	0.35423	0.06391	5281.5	2.6720	0.001
	Ptisana attenuata	Ptis att	0.41377	0.05955	4921.0	2.6409	0.001
	Tristaniopsis glauca	Trist gla	0.46934	0.05557	4592.1	2.6177	0.001
	Grevillea gillivrayi var. gillivrayi	Grev gilvar. gil	0.51497	0.04563	3771.2	2.2579	0.001
	Basselinia gracilis	Bass gra	0.54833	0.03336	2757.2	1.6989	0.005
	Costularia comosa	Cost com	0.58142	0.03308	2734.1	1.7388	0.006
	Melaleuca cf. gnioides	Mela cf. gni	0.64270	0.02837	2344.5	1.5879	0.038
	Gleichenia dicarpa	Glei dic	0.66972	0.02702	2233.4	1.5546	0.023
	Myrsine oblanceolata	Myrs obl	0.69478	0.02506	2071.3	1.4781	0.042
**Fungi**	*Tristaniopsis glauca*	Trist gla	0.07480	0.07480	10146.0	2.4253	0.001
	Grevillea gillivrayi	Grev gil	0.13965	0.06486	8797.7	2.1861	0.001
	Codia spatulata	Codia spa	0.19691	0.05726	7767.6	1.9965	0.001
	Nothofagus aequilateralis	Noth aeq	0.24920	0.05229	7093.3	1.8805	0.001
	Pancheria sp1	Panc sp.1	0.30141	0.05221	7081.8	1.9430	0.001
	Costularia comosa	Cost com	0.34320	0.04179	5668.5	1.5906	0.001
	Meiogyne tiebaghiensis	Meio tie	0.42162	0.03705	5026.4	1.4735	0.010
	Tristaniopsis guillainii	Trist gui	0.45766	0.03604	4888.9	1.4620	0.007
	Austrobuxus ellipticus	Aust ell	0.49222	0.03457	4688.7	1.4295	0.022
	Garcinia amplexicaulis	Garc amp	0.52519	0.03297	4472.6	1.3888	0.039
