# Supplementary material for: Environmental and Geographical Factors Structure Soil Microbial Diversity in New Caledonian Ultramafic Substrates: A Metagenomic Approach
Source: PLoS One. 2016 Dec 1;11(12):e0167405. doi: 10.1371/journal.pone.0167405 (PMC5131939; doi:10.1371/journal.pone.0167405)
Supplement: S6 Table — The R2 that corresponds to the proportion of explained variation in the model by sequentially adding environmental variables, the proportion of variance explained for each variable, the sum of squares (SS), the pseudo-F statistic (analogous to Fisher’s F test) and the P-value are presented. (PDF) [file pone.0167405.s013.pdf]

	Variable	Abbreviation	R^2	% variance explained	SS (trace)	Pseudo-*F*	*P* -value
**Bacteria**	Exchangeable Sulphur	S	0.19583	0.19583	16184.0	7.3055	0.001
	Percentage Hydrogen exchangeable (based on CEC)	H.base	0.27560	0.07977	6592.2	3.1933	0.001
	Exchangeable Zinc	Zn	0.34427	0.06868	5675.6	2.9325	0.001
	Percentage of clay	clay	0.40966	0.06539	5403.8	2.9906	0.001
	Total Iron	Fe.tot	0.45154	0.04188	3461.0	1.9853	0.001
	Exchangeable Manganese	Mn	0.48539	0.03386	2798.0	1.6448	0.004
	pH	pH	0.51739	0.03200	2644.6	1.5913	0.009
	Total Silica	Si.tot	0.54734	0.02994	2474.5	1.5213	0.012
	Ratio Calcium/Magnesium	ratio Ca:Mg	0.57808	0.03074	2540.7	1.6030	0.012
	Percentage of sand	sand	0.60736	0.02929	2420.3	1.5663	0.010
**Fungi**	Exchangeable Sulphur	S	0.07767	0.07767	10535.0	2.5262	0.001
	Total Iron	Fe.tot	0.13559	0.05792	7857.3	1.9433	0.001
	Total Calcium	Ca.tot	0.18843	0.05285	7168.5	1.8232	0.001
	Exchangeable Zinc	Zn	0.23683	0.04840	6565.4	1.7123	0.001
	Total Silica	Si.tot	0.28031	0.04348	5897.8	1.5707	0.005
	Percentage of sand	sand	0.32182	0.04151	5631.0	1.5302	0.004
	pH	pH	0.35853	0.03671	4979.4	1.3734	0.017
	Exchangeable Magnesium	Mg	0.39550	0.03697	5014.4	1.4065	0.012
	Organic Matter	OM	0.43077	0.03528	4785.2	1.3634	0.031
	Exchangeable Manganese	Mn	0.46527	0.03450	4679.3	1.3547	0.045
